# Cultural blind spots: Identifying hidden psychosocial hazards in the workplace

**DOI:** 10.1002/hpja.900

**Published:** 2024-07-11

**Authors:** Theaanna Kiaos

**Affiliations:** ^1^ The Discipline of Psychiatry and Mental Health, School of Clinical Medicine, Faculty of Medicine and Health University of New South Wales Sydney New South Wales Australia

**Keywords:** employee mental health, organisational ethnography, psychosocial hazards, work health and safety

## Abstract

**Issue Addressed:**

This article presents a framework to identify hidden psychosocial hazards and emerging mental health risks in the workplace, thereby assisting Persons Conducting a Business or Undertaking. The framework adds value to the processes outlined in SafeWork NSW's Code of Practice for Managing Psychosocial Hazards At Work. Specifically, the article documents a framework to analyse microcultures and back stage sites of enactment where psychosocial hazards and risks may be hidden or obscured in workplace settings.

**Background:**

The article's framework aims to bring to the surface both the intra and interpersonal tensions employees experience in the social reality they inhabit while they perform their work, thereby positively contributing to organisations and PCBUs by helping them create healthy workplace cultures and psychological safety.

**Methods:**

Specifically, the article discusses partnering with an organisational ethnographer when a PCBU embarks upon psychosocial investigations to: gain access, select employee participants, start conversations, establish rapport, build trust, collect and analyse data.

**Conclusion:**

This article theoretically contributes to health promotion literatures by offering organisations a complementary way of extracting deeper insights and understandings of psychosocial hazards and emerging mental health risks which are not apparent with traditional methods of inquiry.

## 1 INTRODUCTION

In May 2021, SafeWork NSW introduced its Code of Practice for Managing Psychosocial Hazards At Work^i^.^1^ ‘The Code’ is an approved Code of Practice under section 274 of the Work Health and Safety Act 2011 (WHS Act). The WHS Act defines ‘health’ as including both physical and psychological health (p. 4). Specifically, the Code lists 16 psychosocial hazards^ii^ that frequently occur and recur in workplace settings and is claimed to provide practical guidance on how to achieve compliance with the work health and safety standards required under the WHS Act, Work Health and Safety and Safety Regulation (WHO Regulation), including successful techniques to identify and manage psychosocial hazards and risks. As such, this article aims to reflect a complementary resource for organisations and those acting as Persons Conducting a Business or Undertaking (PCBU) by providing a framework for empirical application that brings to the surface both the intra and interpersonal tensions employees experience by breaking down power differentials and by cultivating psychological safety within workplace settings.

According to the Code, psychosocial hazards reflect any part of work that can cause stress and harm to employees while they perform their work. Psychosocial risk exposes the potential of possible harm (death, injury or illness) which might occur if a worker is exposed to a hazard. Consequently, employers are now required to meet legal obligations when managing risks that could lead to potential psychosocial injury. The Code of Practice is admissible in court proceedings under the WHS Act and the WHS Regulation. Employers are responsible to record any identified psychosocial hazard and are legally required to investigate work‐related contributing causes by those who typically hold ‘duty of care’ responsibilities. Duty holders refer to any person who owes a duty under the WHS Act, including a Persons Conducting a Business Undertaking ‘with management and control of a business’ (p. 3). A worker means any person who carries out work for a PCBU, including work as an employee, contractor (or their employee), self‐employed person, outworker, apprentice or trainee, work experience student, an employee of a labour‐hire company placed with a ‘host PCBU or volunteer’ (p. 3). Managers and supervisors are also considered workers.^1^

The Code claims that psychosocial hazards can arise from organisation‐wide systems, work practices and cultural issues. Importantly, the Code specifically states that ‘relevant duty holders should create a positive organisational culture which actively supports early reporting and follow up to ensure psychosocial hazards and risks are managed before serious harm occurs’ (p.24). Furthermore, information about psychosocial hazards should be collected across the organisation. It is highly important to note the Code makes clear that like regulations, codes of practice tend to deal with particular risks and do not cover all hazards or risks that may arise, reflecting a clear area of concern relating to data obscurity in workplaces; an issue this article addresses. Whilst the Code states that compliance with the WHS Act and WHS Regulation may be achieved by following other methods if they achieve an equivalent or higher standard of safety than the one set out in the Code and encourages organisations to be proactive in designing their own compliance protocols, it does not go far enough in assisting PCBUs. Specifically, the Code omits strategies that help identify psychosocial hazards and emerging mental health risks via a thorough analysis of an organisation's various cultures.

## 2 THE CODE'S LIMITATIONS

According to the Code, psychosocial hazard management requires that employers follow a four‐step process. Step one identifies psychosocial hazards by gathering data and closing any knowledge gaps through worker consultation, including the use of survey data. Step two involves assessing psychosocial hazards as presented by the data, thereafter providing a clear course of action that seeks to reduce those hazards. The third step involves controlling psychosocial hazards. Finally, the fourth step involves ‘review and control measures’ (p.17) to ensure employers limit psychosocial hazards and injuries occurring in the future. This article specifically focuses on step one as specified in the Code—the identification of psychosocial hazards by gathering data and closing any knowledge gaps, particularly when organisations undergo significant changes, including ‘downsizing, organisational restructuring, new work arrangements or technologies’, (Ref 1 p.17). Organisational change is known to cause heightened levels of intra‐and interpersonal tension or stress for some employees.^2^

The first step in the Code addresses the risk management process by identifying psychosocial hazards which may arise from the work context or work content. The Code claims the process involves identifying various aspects of work and situations that could potentially harm people and why these may be occurring, including where hazards and risks are, why they are not effectively managed and opportunities to improve the quality of the existing controls. In this regard, the Code states PCBUs should always consider the underlying sources of psychosocial hazards and risks which are likely to be both internal and external to the organisation. To action step one, the Code suggests that PCBUs should consider reviewing organisational documents and records, including but not limited to organisational records and or metrics such as psychosocial hazard and incident reports, complaints and investigations into alleged harmful workplace behaviours, absenteeism, turnover and sick leave data, exit interviews and workers' compensation claims. The Code also states that an analysis of psychosocial hazard and workforce or culture surveys should be adopted. The Code specifically states, ‘If you choose to conduct workplace surveys, giving your workers the option to respond anonymously may improve the response rate and quality of the information you receive. Ideally this information should be collected from a representative cross section of workers or through existing structures such as WHS committees’ (p. 17–18).

The application of cultural surveys are generally considered part and parcel of how managers and leaders monitor their espoused organisational culture,^3^ however cultural surveys also pose significant limitations for capturing valid psychosocial data within the workplace. Culture surveys provide static manifestations of cultural knowledge.^4^, ^5^ Cultural surveys also limit the degree to which senior leaders are able to identify both psychosocial intra‐ and interpersonal tensions that employees experience while they perform their work.^6^ Specifically, cultural surveys do not reveal microcultural particularities such as back stage sites of enactment where employees reflect:

Language and behaviour that is most tightly tied to their underlying feelings … not necessarily reflecting alignment to the organisation's ideology or dominant culture because it has personally worked against them in some form or capacity. Specifically, back stage sites of enactment are used for venting, discussing, highlighting or exaggerating contentious issues within the organisation and matters pertaining to others in relation to themselves,^7^ p. 23.

As the term ‘psychosocial’ suggests, hazards and risks within workplace settings typically relate to people interacting with other people in various contexts. Centering the PCBU's attention on interactions and behaviours between employees at work is therefore logical and necessary. For this reason, this article argues the process suggested by the Code for identifying psychosocial hazards and risks is limited because it is oversimplified and incomplete. This limitation as reflected in the Code could potentially lead to inaccurate or incomplete findings which may result in possible litigation for employers and psychosocial hazards and risks for employees.

## 3 CULTURAL BLIND SPOTS

Firstly, the Code does not acknowledge that cultures within an organisation need to be examined at various layers of analysis to adequately inform stage one of the psychosocial hazard and risk identification process. For instance, Kiaos's^6^, ^7^ research at Service NSW during and after a Machinery of Government (MoG) merger found that microcultures revealed psychosocial hazards and emerging mental health issues in back stage sites of enactment for staff across middle management. In other words, what the Code classifies as workers ‘showing signs of distress’ (p.18). Specifically, the research found that microcultures were shown to function discreetly as a way to protect the interests of parties who functioned within them. Microcultures led to cognitive and emotional tensions between employees and also demonstrated that cultural surveys and focus groups were not optimal tools to identify these tensions. Obtaining detailed understandings of participants' subjective experiences were more easily and accurately obtained by administering in‐depth interviews in unstructured formats.

Pre‐planning was limited from the perspective of participants so they did not have time to think about potential responses to questions in situations which required them to perform on the front stage. In other words, where employees were required to present facades in alignment with the Service NSW ‘DNA’ culture.^8^, ^9^

In‐depth interviews have been shown to enable higher degrees of interpersonal comfort between ethnographers and their participants.^10^, ^11^ By contrast, employees largely do not feel comfortable to freely express their opinions in surveys and focus groups for reasons not limited to issues of power and control, yet they might appear comfortable.^7^ Extant literature has established that culture surveys present severe limitations, not only due to data oversimplification but also the omission of data.^5^, ^12^ Yet another issue posed by the application of cultural surveys reflects controlling survey data by distributing favourable results or a selection of results across an organisation instead distributing all results regardless of how unfavourable they might be. Therefore, quantitative surveys and short answer questionnaires should be used as secondary tools for capturing valid psychosocial hazard and emerging mental health risk data within workplace settings.

The data gathering process is pivotal when investigating and undergoing cultural and psychosocial change within workplace settings. Problematically, if inadequate or incomplete data is captured during stage one of the four stages proposed by SafeWork NSW's Code of Practice, the remaining three stages will plausibly lead to compromised outcomes, with a lack of rigorous, credible and relevant data possibly leading to serious legal ramifications for employers and psychological injuries for their employees. For this reason, identifying psychosocial hazards and emerging mental health risks in workplace settings requires an analysis of microcultures given that intra and interpersonal tensions are more readily observed in back stage sites of enactment. Microcultures function in a way that allows employees to more easily step out of professional character, thereby delivering a level of psychosocial detail not accessible by methods documented in the Code.

## 4 ORGANISATIONAL MICROCULTURES

To identify psychosocial hazards and emerging mental health risks in microcultures, two perspectives of an organisation's culture should first be analysed accurately and thoroughly. Specifically, PCBUs would analyse the integration and differentiation perspectives of an organisation's culture.^5^ The integrationist perspective subverts local values by drawing employees into systems that operate in the interests of corporations. This first step is important because employees across departments, particularly in lower rungs with whom PCBUs would engage may not be completely aware of how they are being used for the organisation's interests and why the chosen language and behaviour of workers reflects the organisation's prescribed social reality on the front stage.^9^ Indeed, some employees may recite organisational rhetoric rather than objectively or critically assess the true nature of their social working environment and their actions that promulgate it (Kunda, 2009). These facts are highly relevant since particular groups are exposed to higher levels of psychosocial risk, such as workers who are younger, older, in training, employees who are new to the organisation, from diverse or intersectional backgrounds or those who have experienced work‐related injuries, illnesses or who have previously been exposed to traumatic events. Such employees must be carefully observed and proactively assisted by PCBUs, if required, to minimise the impact of psychosocial hazards and or emerging mental health risks.

The integration perspective of an organisation's culture perpetuates the prevailing normative ordering within which psychosocial hazards and risks manifest, however does not reveal the full spectrum of psychosocial hazards or emerging mental health risks in workplace settings as power‐centric social realities often lay dormant, unspoken or ignored. According to the Code, some psychosocial hazards will be quite apparent while others require a more comprehensive process to identify them and their underlying causes. Therefore, the second step is to conduct an analysis of an organisation's cultures by adopting the differentiation perspective.^5^ The differentiation perspective becomes particularly valuable as it focuses on cultural manifestations that have inconsistent interpretations. The differentiation perspective empowers PCBUs to conduct a more thorough identification of psychosocial hazards and emerging mental health risks by enabling them to search for dissenting voices within organisational subcultures and microcultures. Hence, the inherent challenge for PCBUs is to bring to the surface which aspects of work are creating psychosocial issues via subcultural analyses by carefully examining different departments, business units and membership groups of an organisation, for instance, leadership teams, middle managers and frontline employees. Indeed, the Code suggests ‘this can be done through consultative forums, workplace surveys, health and safety committee meetings and routine or dedicated project meetings between workers and their supervisor’ (p.17). However, as mentioned, this approach does not guarantee that employees will speak out about psychosocial hazardous aspects of their work, particularly if there are complex power‐centric social realities at play within departments or within organisational memberships groups.

Whilst, the Code suggests that psychosocial hazards and risks can also be identified by PCBUs by conducting ‘walk through/talk through with workers’ in other words, engaging with staff about ‘how work is done under typical operating conditions such as during peak workloads and under expected circumstances, including emergencies (p.18), the Code does not explicitly provide adequate advice about how to expose challenging power differentials between employees and the problems that manifest under such constraints, hence presenting PCBUs with data collection limitations. Such realities are more likely to reveal themselves via taking a third step, that is, by examining microcultures via back stage sites of enactment. PCBUs must understand the complexities involved in conducting an analysis of this sort. To illustrate, clear distinctions between insider and outsider concepts^13^ in the course of identifying psychosocial hazards and emerging mental health risks must be considered by PCBUs as outlined below.

In cultural analysis, taking an insider perspective focuses upon the language that is used by employees as they describe their workplace social reality.^14^ It involves descriptive and interpretive properties by exposing implicit meanings that are held by employees about an organisation's social reality. For this reason, insider information is considered factual and therefore central to an ethnographic investigation. Said differently, what employees think, feel, do and say, in context is recorded and interpreted by the PCBU. Accurate insider interpretations are immensely important in the course of identifying psychosocial hazards and emerging mental health risks because no understanding of an organisation's social reality is valid without representation of employee voices, reflective of their subjective viewpoints and experiences, ideally in situ. For this reason, one central component of the PCBU's role is to account for what really goes on in organisations, in living colour, because workers express themselves in complex ways precisely because they ascribe meaning to various workplace stimuli in different ways. In this case, PCBUs must learn microcultural territory both broadly and locally along with contexts and meanings that shape the sociocultural environment which employees inhabit. PCBUs do this by adding critical questions to uncover psychosocial hazards and emerging mental health risks that have either been previously suppressed, hidden or ignored. PCBUs must examine the power constructs that hold such patterns in place and how employees accept or struggle against these dimensions of organisational life. Once PCBUs add a critical lens to their investigations by examining dimensions of power, the critical psychosocial hazard and emerging mental health risk landscape is illuminated, whereby the interests of parties involved may often reflect power disparities and inequalities.

By contrast, outsider concepts are drawn from pre‐existing theory. Methodologically, this approach examines organisational culture by those who are considered outsiders, such as consultants, usually by adopting survey questionnaires. Interpretations of the data at this level of abstraction negates the possibility of providing deeper insights concerning employees in the context of microcultures via back stage sites of enactment which reveal hidden psychosocial hazards and emerging mental health risks. Therefore, by taking an insider approach, PCBUs would come to appreciate the complexity of patterns of meaning reflected through the analysis of language and behaviour between employees. These insights would concurrently make focusing on the relationship of a few psychosocial hazard and risk variables such as sick leave data, EAP phone calls, employee turnover and excessive annual leave look superficial at best and potentially misleading at worst. For this reason, PCBUs should focus on the question of interest, ‘what is really going on here?’ Said differently, PCBUs should not exclusively focus on policies, procedures, standards or easily accessible data or information that would make the organisation look more competent in assessing and managing psychosocial hazards and risks.

In short, to provide a broad view of the steps required to identify psychosocial hazards and emerging mental health risks in workplace settings through an analysis of an organisation's microcultures, PCBUs must observe the activities of employees, talk with staff willing to answer questions in an informal manner about their working lives, collect artefacts, including texts of various sorts typically consumed by employees and then devise several ways of keeping a comprehensive, detailed and legible record of this information for interpretative purposes.

## 5 IDENTIFYING PSYCHOSOCIAL HAZARDS AND EMERGING MENTAL HEALTH RISKS

This article presents key steps required to conduct a basic critical ethnographic analysis in search for hidden psychosocial hazards and emerging mental health risks in organisations. The key steps involve delineating the role of PCBUs, including gaining access and starting conversations with employees, establishing rapport and building trust, identifying and understanding psychosocial hazards and emerging mental health risks, collecting and analysing data, ensuring rigour, credibility and relevance of the data and managing presenting challenges.

### 5.1 Analysing microcultures through partnership: PCBUs and organisational ethnographers

Organisational ethnographers are increasingly gaining the attention of corporate leaders as well as Human Resource and People and Culture personnel to better understand their employees.^15^ It is advised that PCBUs partner with an organisational ethnographer before and during significant organisational change, given that some employees are likely to experience heightened psychosocial hazards and risks as well as emerging mental health concerns more so during times of change. It is also recommended that officers of the PCBU or workers of a PCBU be guided by an organisational ethnographer to ensure the evidence garnered is objective. The investigation requires an objective subject matter expert skilled in applying critical ethnographic methods who can guide PCBUs during the investigative process.

The organisational ethnographer acts in the best interest of employees while maintaining neutrality and reflexivity during the data collection and interpretation phases of the psychosocial hazard and risk identification process. On this basis, the PCBU gains access to employees through partnering with an organisational ethnographer in an effort to learn about employees as an insider. This process is pivotal in garnering both implicit and explicit meanings employees attribute to the social reality of their workplace. Hence, during the course of the investigation, PCBUs and organisational ethnographers are faced with the paradox of professional distance and personal involvement while collecting data.

This partnership also requires that both PCBUs and organisational ethnographers adopt high degrees of reflexivity to ensure internal validity of the data and to minimise any distortion in the data. In fact, PCBUs and organisational ethnographers must recognise themselves as data because bias is a distinctly human trait,^16^ a point revisited in later sections of this article. To elaborate, PCBUs and organisational ethnographers in search for psychosocial hazards and emerging mental health risks must aim to conduct themselves like an employee within the workplace context they are investigating, rather than reflecting their current position. For instance, if a PCBU or organisational ethnographer is seeking to investigate psychosocial hazards and emerging mental health risks in frontline centres, they should adopt a role as a frontline worker for a portion of the investigation. Role immersion is a critical component to a thorough ethnographic investigation. Specifically, the organisational ethnographer's role is to guide the data collection process while the PCBU takes up their investigative position as a frontline worker. One way that organisational ethnographers guide PCBUs is by catalysing where and how PCBUs should focus their investigative energies. The premise to this insider vis‐à‐vis outsider status means the PCBU's major objective is to determine how to present their affiliation without lying or omitting the truth, while concurrently countering negative assumptions that employees may make about them or other employees within the organisation.

PCBUs would start their work by ensuring they create space for employees to talk and act freely in their own way during the course of interaction. Importantly, PCBUs would not immediately classify data they collect from employees in terms of prior theoretical concepts. In other words, PCBUs should avoid outsider concepts and take an insider approach to inquiry. PCBUs would see data as containing their own patterns and their own concepts and should view analysis as an effort to figure out what those concepts might represent in the social reality in which they are expressed by employees. The final results could overlap onto previous outsider based concepts or theories, but this may not always transpire. New insider concepts based on accurate interpretations of the data would in fact bring PCBUs closer to the psychosocial world in which employees work. This view is also supported by the Code, ‘workers generally know which aspects of work are creating or likely to create hazards or risks and may have practical suggestions on how to manage these’ (p.17).

### 5.2 Gaining access and starting conversations

Before PCBUs engage employees for data collection purposes, they might deliberate upon an appropriate starting place for dialogue. The objective is to learn about accounts of working life from employees' point of view. Hence, it makes sense to start with employee descriptions of social situations at work by gaining an initial characterisation of a range of workplace situations. To do this, PCBUs might use interviews revolving around employees' daily routines or asking for a history of their time in their role at the organisation. As such, PCBUs begin to learn what sorts of social situations exist and how they are interpreted by employees. PCBUs must remember at the outset that critical ethnography is a privilege because it carries with it the responsibility of ensuring accurate interpretation of employees’ accounts of their psychosocial reality.

### 5.3 Establishing rapport and building trust

Perhaps the most valuable component of PCBUs' work will be the process of establishing rapport and building trust with employees. The Code formalises the consultation process with employees by ‘sharing information, giving affected workers reasonable opportunities to express views, taking those views into account before making decisions … and advising workers on the consultation's outcomes in a timely and appropriate manner’ (p.20). As exemplified thus far, this article argues that PCBUs must come to understand psychosocial hazards and emerging mental health risks that employees are faced with by different means. PCBUs would not seek to exclusively consult, but rather to learn directly from employees about how they interpret various psychosocial stimuli, constrained by the various cultures for which they must act, function and transverse within. One central pillar to successful PCBU engagement is to approach the task as informally as possible.

PCBUs may start by expressing interest and reflecting back to employees their statements once they begin giving accounts of psychosocial hazardous events by adopting their language and where appropriate, their behaviour. This approach then enables PCBUs to use a variety of strategies to garner more detail reflecting the accounts of employees, including by formulating questions based on what employees are saying and doing. In this respect, PCBUs move much further beyond consultation and therefore beyond the formalised steps as stipulated by the Code. To focus exclusively on consultation would be to miss the point of adopting critical ethnographic methods that are centred on both the value of language and behaviour that employees adopt as they traverse various cultural stimuli. Furthermore, to focus exclusively on consultation would neglect the opportunity to learn how relationships are established between PCBUs and their employees by translating language and behaviour into insights that reveal patterns of subjective meaning generated by the social reality employees inhabit. In other words, new psychosocial knowledge. This process involves the collection of raw material reflecting the daily activities of employees. The only way to access those activities is to fully establish relationships with employees, to participate with them in activities they do and by observing and interpreting what is going on in such contexts. Once trust has been built, observation and informal interaction with employees makes it possible for information to be safely released from a psychological perspective.

### 5.4 Identifying and understanding psychosocial hazards and emerging mental health risks

Once trust has been built and guided by the partnering organisational ethnograher, PCBUs would aim to identify, hone in and understand psychosocial hazards and emerging mental health risks for employees. To reiterate, in back stage sites of enactment, employees reflect language and behaviour that is most tightly tied to their underlying feelings and are used by employees to manage feelings associated with intra‐ and interpersonal tension that has been caused by the organisation. To interpret the collected data, PCBUs must manage ‘rich points’ and analyse ‘strips of information’ gathered by employees. Both activities present PCBUs with opportunities to explore previously unchartered terrain, enabling PCBUs to bring insights to the surface for further investigation.

According to Agar^17^ ‘rich points’ (p.31) reflect language and or behaviour that is typically unexpected and therefore requires further deliberation before an interpretation is made. In other words, a rich point reflects a breakdown in knowledge translation between PCBUs and their employees. For instance, it would be easy for the PCBU to make an assumption about the psychosocial organisational environment in relation to employees' subjective experiences, rather than to further deliberate upon why the workplace context has evolved as it has and why employee subjective experiences were expressed, both spatially and temporally in such ways. PCBUs would use rich points as an opportunity to further investigate microcultural contextualities, particularly the type of language and behaviour used, in context, by informally adopting a series of open‐ended questions until all lines of inquiry have been explored, are clear, assumptions transcended and have led to some sense of coherence. This approach would ensure that employees have confirmed all identified rich points and agree on their meanings, thus restoring knowledge translation. PCBUs would then translate each rich point in a way that is both practical and useful during steps 2, 3 and 4 of the Code which follow thereafter. ‘Strips of information’, (Ref 17 p.33) on the other hand reflect ethnographic segments of data. Strips of data may include interviews or sequences of behaviour encountered by PCBUs. The PCBUs' value in this process is made evident when they successfully identify and understand psychosocial hazards and emerging mental health risks that have been hidden from view and therefore have remained unexplored through their interviews and recordings of observed behaviour. The PCBUs greatest value however is made evident when they have coherently expressed these psychosocial hazards and emerging mental health risks to inform interventions which minimise or remove them from causing harm to employees, thereby also preventing litigation.

### 5.5 Collecting and analysing the data

It is recommended PCBUs commence the analytic process by coding strips of information that focus on the same topic. For instance, PCBUs may code statements at different levels of generality, topics and subtopics until the most micro of topics have been coded. PCBUs may also find there are clear transitions, from one topic to another which should also be coded, but only if they seem relevant to the psychosocial hazard or emerging mental health risk that was unearthed during interviews and behavioural observations. As PCBUs move through their collected data, they may begin to notice that some topics recur and may hold valuable information concerning an unidentified psychosocial hazard or emerging mental health risk. These recurrent themes are prime candidates for categorisation and coding. As such, PCBUs will need to give themes a suitable name and progress through the observational and transcript data again to mark which sections fit the newly named theme, that is, the newly identified psychosocial hazard or mental health risk.

The PCBU's goal in this process is to devise ways of coding newly identified psychosocial hazards and emerging mental health risks that cover as much of the observational data and transcribed communication between employees as possible. Once the PCBU has categorised all the material, they would then categorise sections of those transcripts according to that particular psychosocial hazard or emerging mental health risk. Each psychosocial hazard or emerging mental health risk can then be read back to check for consistency by each employee who took part in the investigation, ensuring the identified psychosocial hazard or risk reflects rigour, credibility and relevance in the context in which it was discovered. Post the investigation, the PCBU would also create additional informal questions to ask employees to ensure PCBUs have interpreted the data accurately. By doing so, PCBUs confirm the organisation's various cultures for which psychosocial hazards and emerging mental health risks have transpired, thereby enriching the knowledge content of the organisation by learning more about its implicit psychosocial environment and pockets of the organisation where microcultures might emerge in the future. As a result, PCBUs are equipped to identify, understand, translate knowledge and act in ways that make sense from employees' point of view and present that information to decision makers who can initiate cultural change.

### 5.6 Rigour, credibility and relevance

Firstly, it is important to mention that unlike quantitative methods that consider validity, reliability and empirical generalisability important, these concepts are not applicable when conducting ethnographic investigations. However, terms including rigour, credibility and relevance are considered highly important concepts.^18^ Rigour is described as data appropriateness and thoroughness. Credibility relates to meaningful and well‐presented findings. Finally, relevance is the utility of findings which are used to judge the quality or trustworthiness of the data. In short, PCBUs would seek to present a rigorous, credible and relevant account of an organisation's psychosocial hazards and emerging mental health risks by utilising a specific set of criteria and by emphasising the applied methodological techniques used to analyse and present the data for the PCBUs' intended audience.

In the context of identifying psychosocial hazards and emerging mental health risks in workplace settings, it is also important to briefly discuss matters concerning both external and internal reliability. Since psychosocial situations cannot precisely be reconstructed for the purpose of data replicability, it is important to enhance external reliability of the collected data by recognising the PCBUs' position, the organisational ethnographer's position as well as how employees are screened and selected for analysis as part of the investigation. For this reason, PCBUs should liaise with their organisational ethnographer to help delineate the type of employees who should serve as participants in the investigation and the decision processes involved in selecting their participation, as well as the social situations and conditions for which both PCBUs and employees find themselves in. Employee selection might depend upon factors that include both ‘accessibility’ and ‘willingness’ as well as ‘knowledge, insight and skill’, (Ref 19 p.89). It is important to note that some employees may reveal information in some contexts and circumstances and not in others. In such circumstances, PCBUs should continuously cross‐check information they acquire and the interpretations they develop by way of revisiting topics or themes previously explored with employees.

Relatedly, internal reliability reflects the degree to which other PCBUs would match them with their own data in the same way as did the original PCBU. Some scholars believe ethnographic accounts cannot be considered internally reliable because time is not a static phenomenon and spatial dimensions change over time, hence, data insights would likely change too.^20^ To manage the complexities relating to internal reliability, PCBUs may also enlist the aid of other PCBUs ‘to confirm what the observer has seen and recorded’, (Ref 20 p.42). In addition, PCBUs could record verbatim accounts reflecting what employees say as well as narratives of behaviour and activity.^20^ Interpretative comments may be added, deleted or modified; however it is imperative to record precisely who said and did what and under what circumstances.^20^, ^21^ As guided by their organisational ethnographer, PCBUs would record highly descriptive narratives by adopting a thick descriptive approach,^22^ reflecting accurate accounts of employees. PCBUs may also use a variety of mechanical devices to record and preserve their data. For instance, audio recorders are considered standard equipment in the collection of ethnographic data. Indeed, PCBUs are encouraged to use a variety of equipment and a wide range of techniques to supplement and corroborate their findings.

### 5.7 Presenting challenges

One further matter concerning internal validity again points to the insider vis‐à‐vis outsider relationship and the blurring of professional boundaries while PCBUs conduct their investigations. Specifically, the dynamics between the overt status of the PCBU in relation to employees may change since the relationship has blurred boundaries and requires continual negotiation and renegotiation with employees. Furthermore, the presence of the PCBU can influence employee behaviour, changing the way they perform or act during the investigation known as the ‘observer effect’, (Ref 23 p.357), reflecting another reason why partnering with an organisational ethnographer is vital, that is to preserve the integrity of the data.

Observer effects occur when employees become dependent on PCBUs for personal and professional status enhancement or the satisfaction of psychological or sociological needs. In such cases, a symbiotic relationship may develop between PCBUs and employees, leading to what some scholars believe causes transference.^20^ Briefly, the concept of transference is one where an interpersonal experience precludes obtaining accurate data because the data is distorted by what employees perceive is a ‘special relationship’. Therefore, PCBUs must manage transference carefully by avoiding ‘problems of entanglement by assuming a position of neutrality’, (Ref 20 p.46). For instance, employee detachment from a PCBU could destroy rapport and cause other employees to infer indifference or even hostility toward them. In addition, employees ‘may lie, omit relevant data, or misrepresent their claims’, (Ref 20 p.46). This may be a consciously planned performance in ‘which subjects seek to reveal themselves in the best possible light, or it may be an unconscious distortion performed to provide what participants believe the researcher wants to see’, (Ref 20 p.46). In order to mitigate possibilities of transference, PCBUs must carefully disengage from bonds where they suspect that employees are distorting claims.

Another presenting challenge might reflect what psychosocial hazards and emerging mental health risks should be changed and how and the direction which solutions lie. Yet another challenge reflects false consciousness: under what conditions do employees suffer from false consciousness and when are they right? Supported by the techniques discussed in this article, critical ethnographic investigations are designed to unearth psychosocial phenomena of this nature. In short, PCBUs cannot escape the responsibility nor requirement to carefully manage presenting challenges while undergoing their investigations. PCBUs must be prepared to ask themselves difficult questions in order to deliver worthwhile solutions, knowing that each solution presents practical consequences for some employees more so than others.

## 6 CONCLUSION

This article assists PCBUs by adding value to the processes outlined in SafeWork NSW's Code of Practice for Managing Psychosocial Hazards At Work. The article's framework is designed to identify hidden psychosocial hazards and risks as well as emerging mental health issues, aiming to bring to the surface both the intra and interpersonal tensions employees experience in the social reality they inhabit while they perform their work. The article proposes that limitations are presented in step one of the Code—identifying psychosocial hazards by gathering data and closing knowledge gaps through worker consultation within SafeWork NSW's Code of Practice: Managing Psychosocial Hazards at Work. The Code states that survey data is recommended to close knowledge gaps, however this article strongly suggests that surveys pose PCBUs with severe knowledge limitations. The article therefore purports that PCBUs should thoroughly investigate an organisation's various cultures to identify hidden psychosocial hazards and emerging mental health risks. By doing so, PCBUs will limit psychosocial injuries occurring to their employees while minimising the risk of litigation. Specifically, this article provides an alternative method to gain deeper understanding of employees’ experience of work and any potential hazards and risks to their psychological health. The article builds a case for PCBUs to work with organisational ethnographers to analyse the integration and differentiation perspectives^5^ of an organisation's culture by adopting critical ethnographic methods. Specifically, by analysing the differentiation perspective, PCBUs gain access to microcultures that reveal back stage sites of enactment where psychosocial hazards and emerging mental health risks are often hidden or obscured in workplace settings.

In summary, by adopting the framework proposed in this article, PCBUs would aim to search for employee dissent by identifying psychosocial hazards and emerging mental health risks that lay behind implicit constraints of power and control with a particularly strong focus upon marginal members of the occupational community, including employees who are younger, older, in training, employees who are new to the organisation, from diverse or intersectional backgrounds or those who have experienced work‐related injuries, illnesses or who have previously been exposed to traumatic events who might be at higher risks to experience mental health issues at work. As such, this article theoretically contributes to health promotion literatures by offering organisations with a complementary framework to SafeWork NSW's Code of Practice for Managing Psychosocial Hazards At Work, offering PCBUs an opportunity to obtain deeper insights reflective of psychosocial hazards and risks within workplaces which are not apparent with traditional methods of inquiry.

## CONFLICT OF INTEREST STATEMENT

The author declares that they have no conflict of interest.

## ETHICS STATEMENT

This article does not require ethics approval.

## Data Availability

Data sharing is not applicable to this article as no new data were created or analyzed in this study.

## Endnotes

^i^
See: https://www.safework.nsw.gov.au/resource-library/list-of-all-codes-of-practice/codes-of-practice/managing-psychosocial-hazards-at-work.

^ii^
See Appendix A: Table A1.

## APPENDIX A

**TABLE A1:** Common psychosocial hazards.

Hazard	Example
Role overload (high workloads or job demands)	• Too much to do in a set time or with insufficient workers or resources • Unachievable task deadlines, expectations or responsibilities • Unpredictable shifts or hours of work, shift structures or rosters that do not allow adequate time for workers to recover • Frequent cognitively difficult work • Multiple tasks that require repeated rapid switching between each to complete them, so it is difficult to concentrate • Where there is sustained or frequent exposure to emotionally distressing situations • Tasks that require workers to continually show false displays of emotion, e.g., customer service roles • Tasks and decisions that are safety critical and that may have a serious impact on health and safety of workers and others.
Role underload (low workloads or job demands)	• Tasks or jobs where there is routinely too little to do • Highly repetitive or monotonous work (like picking and packing products, monitoring production lines).
Exposure to traumatic events	Where workers provide care to those experiencing a traumatic event or listen to, view or read detailed descriptions of harrowing and traumatic events experienced by others, including • Emergency responders or health‐care workers • Rape crisis and child protection workers, officers of the course, lawyers or immigration officers and so forth • Experiencing, witnessing, or investigating a serious near miss, injury or workplace fatality.
Role conflict or lack of role clarity	• Conflicting priorities within roles (e.g., providing ‘good customer service’, but with insufficient time allowed to spend with customers) • Uncertainty around roles, tenure, tasks and work schedules and standards (frequent changes, lack of clear explanations to role, or tasks, important task‐related information/training is not available, or not providing a clear performance agreement or reporting requirements)
Low job control	Where workers: • Havel little control over how they do their work, when they can change tasks or take breaks • Are not involved in decisions that affect their work or clients • Are unable to speak up about WHS and the way work is done. Note: where the safety or quality requirements needs to be strictly prescribed, there is likely to be more limited opportunities for worker input into decision making, however workers must still be consulted.
Conflict or poor workplace relationships between workers and their supervisors and managers and co‐workers	Where there are: • Frequent disagreements about how work should be done • Frequent interpersonal workplace conflict • Harmful workplace behaviours Conflict can be stressful, especially when the consequences of miscommunication can lead to additional time pressures or serious errors. Conflict can escalate if it is not dealt with promptly and fairly and if steps are not taken to address poor, unacceptable or harmful behaviours.
Poor support from supervisors and managers	Where there is: • Inadequate information, advice and help with work tasks or to resolve issues, or access to necessary equipment and resources • Performance feedback or other management action which is unreasonable or delivered in an unreasonable manner.
Poor co‐worker support	Where there is: • Inadequate information, advice and help to complete tasks and support for work‐related matters from co‐workers.
Workplace violence	Including, by workers, clients, patients, visitors or others see (add ref)
Bullying	Incidents of bullying by workers, clients, patients, visitors or others See (ref)
Harassment including sexual harassment	Single or repeated incidents of forms of harassment by co‐workers, clients, patients, visitors or others around a person's race, religion, gender, age, disability, etc. See (ref)
Inadequate reward and recognition	Where: • Workers efforts are not recognised • There are not reasonable opportunities for skills development and fair career advancement—UNSW professional staff
Hazardous physical working environments	Where there is exposure to hazardous physical working environments to the extent that it evokes a physiological or stress response. For example, due to concerns about exposure to biological or chemical agents where there is inadequate personal protective equipment (PPE).
Remote or isolated work	Arising from the location, time or the nature of the job. For example: • Where there is limited access to other people, reliable communication or technology to get physical and emotional support, if required. • Working alone or in other peoples’ homes if accessing help, especially in an emergency is difficult (such as, where there is potential exposure to violent or aggressive behaviour).
Poor procedural adjustment (processes for decision making)	Where there are absent or inadequate, unfair or inconsistent application of the processes, for example: • Applying organisational policies or procedures (for example, fair access to preferred shifts or overtime) • Allocating work and resources, or • Managing job performance
Poor organisational change consultation	Where there is: • Poor consultation or communication with other relevant duty holders or affected workers about significant changes • Insufficient consideration of the impact of changes on WHS and performance • Poor practical support for affected workers during change implementation
